# The established chest MRI score for cystic fibrosis can be applied to contrast agent-free matrix pencil decomposition functional MRI: a multireader analysis

**DOI:** 10.3389/fmed.2025.1527843

**Published:** 2025-03-18

**Authors:** Eva Steinke, Grzegorz Bauman, Ingo G. Steffen, Isabel T. Schobert, Stephanie Thee, Zulfiya Syunyaeva, Jobst Roehmel, Helena Posch, Ute L. Fahlenkamp, Carolin Scale, Simon Veldhoen, Oliver Bieri, Mark O. Wielpütz, Marcus A. Mall, Mirjam Stahl, Felix Doellinger

**Affiliations:** ^1^Department of Pediatric Respiratory Medicine, Immunology and Critical Care Medicine, Charité – Universitätsmedizin Berlin, Berlin, Germany; ^2^German Center for Lung Research (DZL), Berlin, Germany; ^3^Berlin Institute of Health (BIH) at Charité, Berlin, Germany; ^4^German Center for Child and Adolescent Health (DZKJ), Berlin, Germany; ^5^Division of Radiological Physics, Department of Radiology, University of Basel Hospital, Basel, Switzerland; ^6^Department of Biomedical Engineering, University of Basel, Basel, Switzerland; ^7^Department of Pediatric Hematology and Oncology, Charité - Universitätsmedizin Berlin, Berlin, Germany; ^8^Department of Radiology, Charité - Universitätsmedizin Berlin, Berlin, Germany; ^9^Department of Radiology, Division of Pediatric Radiology, Charité - Universitätsmedizin Berlin, Berlin, Germany; ^10^Department of Diagnostic and Interventional Radiology, Heidelberg University Hospital, Heidelberg, Germany; ^11^Translational Lung Research Center (TLRC), German Center for Lung Research (DZL), Heidelberg, Germany; ^12^Department of Diagnostic and Interventional Radiology with Nuclear Medicine, Thoraxklinik at University of Heidelberg, Heidelberg, Germany; ^13^Department of Diagnostic Radiology and Neuroradiology, University Medicine Greifswald, Greifswald, Germany

**Keywords:** magnetic resonance imaging, matrix pencil decomposition, cystic fibrosis, quantitative imaging, functional imaging

## Abstract

**Background:**

Established morpho-functional chest magnetic resonance imaging (MRI) detects abnormalities in lung morphology and perfusion in people with cystic fibrosis (pwCF) using a dedicated scoring system. Functional assessment is performed using contrast-enhanced (CE) perfusion MRI. Novel matrix pencil decomposition MRI (MP-MRI) is a contrast agent-free alternative, but further validation of this technique is needed.

**Objectives:**

The aim of this study was to evaluate the applicability of the validated morpho-functional chest MRI score for CE perfusion and MP perfusion MRI in a multireader approach.

**Methods:**

Twenty-seven pwCF (mean age 20.8 years, range 8.4–45.7 years) underwent morpho-functional MRI including CE perfusion and MP perfusion MRI in the same examination. Nine blinded chest radiologists of different experience levels assessed lung perfusion and applied the validated chest MRI score to CE- and MP-MRI. Inter-reader agreement of perfusion scores in CE- and MP-MRI were compared with each other and with the MRI morphology score. Differences according to the readers’ experience were also analyzed.

**Results:**

The CE perfusion scores were overall lower than the MP perfusion scores (6.2 ± 3.3 vs. 6.9 ± 2.0; *p* < 0.05) with a strong correlation between both perfusion scores (*r* = 0.74; *p* < 0.01). The intraclass correlation coefficient (ICC) as measure for inter-reader agreement was good and significant for both perfusion scores, but higher for the CE perfusion score (0.75, *p* < 0.001) than for MP perfusion scores (0.61, *p* < 0.001). The Bland–Altman analysis revealed a difference in CE and MP perfusion scores with more extreme values in CE perfusion scores compared to MP perfusion scores (*r* = 0.62, *p* < 0.001). The morphology score showed a moderate to good correlation with the CE perfusion score (*r* = 0.73, *p* < 0.01) and the MP perfusion score (*r* = 0.55, *p* < 0.01). We did not find a difference in scoring according to the radiological experience level.

**Conclusion:**

The established chest MRI score can be applied both to validated CE and novel MP perfusion MRI with a good interreader reliability. The remaining difference between CE and MP-MRI scores may be explained by a lack of routine in visual analysis of MP-MRI and may favor an automated analysis for use of MP-MRI as a noninvasive outcome measure.

## Introduction

Cystic fibrosis (CF) lung disease is the main cause for morbidity and mortality in people with CF (pwCF) (1–3). CF is caused by mutations in the Cystic Fibrosis Transmembrane Conductance Regulator (*CFTR*) gene that lead to pathologically thickened endobronchial mucus and impaired mucociliary clearance, which favors pulmonary inflammation and infection (4, 5). These pathologies consequently entail progressive structural and functional lung abnormalities in people with CF.

Imaging of the lung is of great importance both in acute exacerbations and in the long-term monitoring of pwCF (6). Chest magnetic resonance imaging (MRI) is a non-invasive and sensitive imaging modality able to detect early morpho-functional abnormalities in pwCF (7–9). Its agreement with findings on computed tomography (CT) and the multicenter-feasibility have been previously demonstrated (8, 10). Chest MRI enables the detection of CF-typical changes from early childhood throughout the entire life and detects disease onset, progression and response to therapy (11–15). Since highly effective CFTR modulator therapies have emerged and led to an increased life expectancy in pwCF, radiation-free imaging techniques such as MRI become more relevant for routine care of pwCF (16, 17). Compared to CT, MRI not only has the advantage of avoiding potentially harmful ionizing radiation, but also offers the possibility of obtaining information about lung function by visualizing regional pulmonary hypoventilation and hypoperfusion (18–21). The validated morpho-functional MRI protocol includes spatially resolved sequences for morphological assessment and temporally resolved first-pass lung perfusion imaging by use of intravenous contrast agent to evaluate lung perfusion (7, 17, 22). The dedicated chest MRI score is a semi-quantitative scoring system designed for the visual assessment of typical MRI findings in CF. It was introduced by Eichinger et al. in 2012 and has repeatedly been validated (7, 17).

The established time-resolved MR angiography records the first passage of a contrast agent bolus through the pulmonary circulation and therefore requires intravenous administration of gadolinium-containing MRI contrast agents (contrast-enhanced perfusion imaging, CE perfusion). This technique is considered the gold standard. In recent years, several new MRI techniques based on Fourier decomposition have been developed and investigated (18, 21, 23, 24). Matrix pencil decomposition (MP)-MRI, a further development of the Fourier decomposition technique, offers greater robustness due to a more precise analysis of cardiac and respiratory signals and an improved image acquisition (19, 21). These novel MRI techniques such as phase resolved functional lung (PREFUL) MRI, self-gated non-contrast-enhanced functional lung (SENCEFUL) MRI and MP-MRI have several advantages over the established CE perfusion MRI: Firstly, the otherwise mandatory administration of gadolinium is no longer necessary; secondly, lung ventilation can be examined in addition to lung perfusion; and thirdly, the data can be analyzed by computer software and provide fully automated quantitative measurements (25).

Currently, we are in a transitional phase in which established, contrast agent-based MRI techniques such as first-pass perfusion imaging are gradually being replaced by innovative functional MRI techniques. In this transitional phase, it is essential to be able to compare CE perfusion MRI with the new techniques. One way to enable a direct comparison is to calculate color-coded lung maps from the MP-MRI data, because human reviewers can then evaluate CE perfusion and MP perfusion MRI using the same visual scoring system.

Since the visual evaluation of MRI depends on a radiologist, any assessment and grading might be subject to variations. Most publications include multiple readers of the same experience level to compare different examination methods (26–28). However, very few research groups have studied radiological assessment by readers of different experience levels. Studies with radiologists of varying experience levels mostly state an improved inter-reader agreement and increased diagnostic accuracy with more experience (28, 29). However, considerable inter-observer variations are well described even independent of the radiological experience levels (30–33).

The aim of this study was therefore to investigate whether the validated and established chest MRI score is suitable for scoring the visualizations of MP-MRI data semi-quantitatively. In addition, we wanted to investigate the influence of a reader’s experience by having radiologists of different experience levels evaluate morphological MRI sequences, CE perfusion MRI and visualizations of MP perfusion MRI in a multireader study design.

## Materials and methods

### Study design and participants

This prospective observational study was approved by the Ethics Committee of Charité - Universitätsmedizin Berlin (EA2/170/20) and informed written consent was obtained from all patients or their legal guardians. During a period of 18 months, a total of 27 pwCF aged 8 years and older in stable clinical condition were prospectively recruited for this study. Patient characteristics are summarized in Table 1. All patients underwent chest MRI as part of their annual routine check-up. Imaging was visually assessed by nine blinded radiological readers with different levels of experience, and a semi-quantitative score was recorded. Readers were divided into three groups according to their level of experience: (I) recently graduated radiology residents with no radiological expertise (0 years), (II) board-certified radiologists with other specialties (intermediate experience), and (III) experts in chest imaging (>5 years of experience in chest MRI of pwCF) with three readers per experience level.

**Table 1:** Clinical characteristics of study population.

Characteristic	Mean ± SD
Number of pwCF	27
Age, years (range)	20.78 (8.39–45.66)
Male / female (%)	15 (55.55) / 12 (44.44)
Body weight, kg	51.0 ± 17.4
Body height, cm	160.0 ± 17.9
BMI, kg/m^2^	19.2 ± 3.7
*CFTR* genotype	
F508del/F508del, *n* (%)	10 (37.1)
F508del/other, *n* (%)	11 (40.7)
Other/other, *n* (%)	6 (22.2)
CFTR modulator therapy	
Lumacaftor/Ivacaftor, *n* (%)	5 (18.5)
Tezacaftor/Ivacaftor, *n* (%)	3 (11.1)
None, *n* (%)	19 (70.4)
Pancreatic insufficiency, *n* (%)	22 (81.5)
*Pseudomonas aeruginosa* infection*, n* (%)	10 (37.1)

Data are presented as mean ± standard deviation, if not indicated otherwise. pwCF, people with cystic fibrosis; MRI, magnetic resonance imaging; BMI, body mass index; CFTR, cystic fibrosis transmembrane conductance regulator.

### Magnetic resonance imaging

All imaging was acquired on one of two different clinical 1.5 Tesla whole-body MR scanners (MAGNETOM Avanto or MAGNETOM Aera, Siemens Healthineers AG, Erlangen, Germany). The standardized morpho-functional MRI protocol was performed as previously described and included T1-weighted sequences before and after intravenous contrast agent administration, T2-weighted sequences, CE first-pass perfusion MRI (7, 17, 22) as well as contrast agent-free functional MP MRI (21, 34–36).

To visualize the morphological lung changes caused by CF, a high-resolution T2-weighted sequence with fat saturation that uses the PROPELLER k-space trajectory method (T2 BLADE, Siemens Healthineers) and a T1-weighted spoiled 3D gradient echo sequence that uses a breath hold technique (volumetric interpolated breath hold examination, VIBE, Siemens Healthineers) were acquired. The T1-weighted sequence was repeated after contrast agent administration. All three morphological sequences were scanned in transversal and coronal plane with a slice thickness of 4.0 mm.

First-pass lung perfusion scans were performed with a spoiled gradient-echo sequence (time resolved angiography with stochastic trajectories, TWIST, Siemens Healthineers) in coronal plane (TE/TR = 0.8/2.05 ms, flip angle = 25°, slice thickness = 5.0 mm, matrix size = 256 × 256). The field of view was adjusted to the patient’s age and was 280 × 280 mm for children below 10 years and 320 × 320 mm for teenagers and adults. The measurement started simultaneously with the i.v. administration of a gadolinium-based contrast agent via a peripheral catheter or central line at a rate of 2–4 mL/s. The contrast agent dose was adjusted according to body weight and aimed for a concentration of 0.5 mmol/kilogram. This resulted in total doses of 1.5–8.0 mL Gadobutrol or 2.3–16.0 mL Gadoteric acid in our cohort. During a breath hold at full inspiration, the first passage of the contrast bolus through the pulmonary circulation was captured within a dynamic series of 30 volume datasets of the whole lung. Each of these datasets represents 1.5 s, resulting in a total acquisition time of CE perfusion MRI of 45 s. When the contrast bolus is injected into a peripheral vein, as is usually done at the back of the hand or in the cubital fossa, maximum contrast in the lung parenchyma usually is reached 20 ± 5 s after injection. This data set, with the strongest pulmonary contrast, must be identified and is then visually assessed for the presence of (regional) pulmonary hypoperfusion.

MP-MRI scans were performed prior to CE first-pass perfusion using two-dimensional time-resolved ultra-fast bSSFP pulse sequence (ufSSFP) (21, 35, 36). Multiple coronal image sets were acquired during 48 s of free-breathing. Scan parameters for ufSSFP were as follows: field-of-view = 400 × 400–450 × 450 mm^2^, matrix size = 128 × 128 interpolated to 256 × 256, slice thickness = 12 mm, echo time (TE) = 0.67 ms, repetition time (TR) = 1.60 ms, bandwidth = 2056 Hz/pixel, flip angle of 65°, GRAPPA factor 2, 160 coronal images per slice. The nominal acquisition time for one image was 120 ms, followed by a waiting time of 180 ms, resulting in a total acquisition time of 300 ms per image and an acquisition rate of 3.3 images per second. In order to cover the whole lung, imaging was performed using between 8 to 10 slices, which resulted in an overall scanning time of 6 to 8 min. During the scan, the examined persons are only required to cooperate passively; they must try not to move and breathe evenly, but they do not have to perform breathing exercises. The color-coded maps of pulmonary perfusion were generated using an automated image analysis software – TrueLung (37).

Both techniques of pulmonary perfusion imaging are acquired in coronal plane, CE perfusion MRI during a breath hold and MP perfusion MRI in free breathing. The primary slice thickness of both methods is different, namely 5.0 mm for CE-MRI and 12.0 mm for MP-MRI, as described above. In both methods, these layers overlap somewhat so that the entire lung is imaged without gaps. In adolescents and adults, a normal data set of the entire lung therefore consists of approximately 30 coronal CE-MRI images and 12–14 coronal MP-MRI reconstruction images.

### Visual MRI assessment

The visual assessment of CF lung disease in this study was based on the validated chest MRI score (7). This scoring system is based on a separate analysis of each individual lung lobe, with the lingula considered as a separate sixth lobe. The morphology score consists of 5 subscores that record the following CF-associated findings: (1) bronchiectasis/wall thickening, (2) mucus plugging, (3) abscesses/sacculations, (4) consolidation, (5) special findings (i.e., pleural findings). The perfusion score is the sixth subscore of the global score, assesses the extent of pulmonary hypoperfusion and thus indirectly reflects the hypoxic vasoconstriction, which is assumed to be the imaging correlate of hypoxic vasoconstriction. In this system, each lobe is scored 0 (no abnormality), 1 (<50% of the lobe affected) or 2 (>50% of the lobe affected) points depending on the extent of the pathology examined for each subscore. Consequently, each subscore ranges from 0 to 12 points and the global score (morphology score + perfusion score) ranges from 0 to 72 points.

All readers were initially trained in the comparison of CE perfusion MRI and MP perfusion MRI on a dataset of 10 pwCF who were not included in this study. Visual analysis of morpho-functional MRI and MP-MRI data was feasible in all 27 pwCF. Nine radiologists with different degrees of experience in the evaluation of chest MRI applied the validated chest MRI score and rated the extent of CF-related morphological changes in T1-weighted and T2-weighted sequences and the size of regional pulmonary perfusion defects in CE perfusion and MP perfusion MRI accordingly (7). T1 without contrast agent, T1 with contrast agent and fat-saturated T2 were each available in transversal and coronal plane. These three sequences were evaluated together to assess the five morphology subscores. This analysis is largely based on the T2 PROPELLER (BLADE) sequence, which many readers even use exclusively. The two T1 sequences can provide additional information for some of the subscores, especially consolidation and special findings (which refers to pleural changes).

Scoring was performed lobe-based with 0 (no abnormality), 1 (<50% of the lobe affected) or 2 (>50% of the lobe affected) points given for each lobe depending on the extent of the examined pathology. Consequently, each subscore ranged between 0 and 12 points. The color-coded MP-MRI lung maps were scored in analogy to the CE perfusion score. The readers assessing CE perfusion MRI and MP perfusion MRI were blinded to the results of the morphological standard sequences described above. All readers assessed all sequences of one type in a row in one session and in a random order of patients. It was considered impossible to trace morphological sequences, CE perfusion MRI and MP perfusion MRI back to the same patient. All 27 MRI examinations were fully scored by the 9 readers, resulting in a total of 243 datasets, each consisting of the MRI morphology score, the CE perfusion score and the MP perfusion score. All readers were blinded to clinical and demographic characteristics.

### Statistical analysis

Statistical analyses were performed with R version 4.0.2 (R Foundation for Statistical Computing, Vienna, Austria). According to histograms and Kolmogorov–Smirnov tests, a normal distribution of metric data was not assumed. Data are presented as mean ± SD, median, interquartile range (IQR; 25th – 75th percentile), and range (min – max) as appropriate. Non-parametric tests such as the Wilcoxon signed-rank test were applied as appropriate. Spearman’s rank correlation coefficient r was used to describe the association between metric parameters. According to *Rowntree* and *Karlik* the correlations are assumed to be negligible (*r* = 0.0–0.2), weak/low (*r* = 0.2–0.4), moderate (*r* = 0.4–0.7), strong (*r* = 0.7–0.9), and very strong (*r* = 0.9–1.0) (38). The intraclass correlation (ICC) was calculated as ratio of variance of interest and total variance using linear mixed effects models whereas differences between readers were calculated by two-way ANOVA (39). A Bland–Altman analysis was used to assess the readers’ agreement and compare the two methods visually. A *p*-value <0.05 was considered statistically significant.

## Results

### Clinical characteristics of study population

The study group consists of 27 pwCF with a mean age of 20.8 (8.4–45.7) years (Table 1). Fifteen male and 12 female pwCF with a mean body weight of 51.0 ± 17.4 kg, a body height of 160.0 ± 17.9 cm and a BMI of 19.2 ± 3.7 kg/m^2^ were included. Ten participants were homozygous for the *F508del* mutation, 11 participants were compound-heterozygous with one *F508del* mutation and six participants had two other mutations in the *CFTR* gene. 81.5% of the pwCF had an exocrine pancreatic insufficiency (Table 1). Five pwCF were treated with the CFTR modulator Lumacaftor/Ivacaftor, three pwCF with Tezacaftor/Ivacaftor while 19 pwCF did not receive causal treatment. *Pseudomonas aeruginosa* was detected in 37.1% of the participants.

### Differences in CE and MP perfusion scores between pwCF and readers

**Figure 1:**
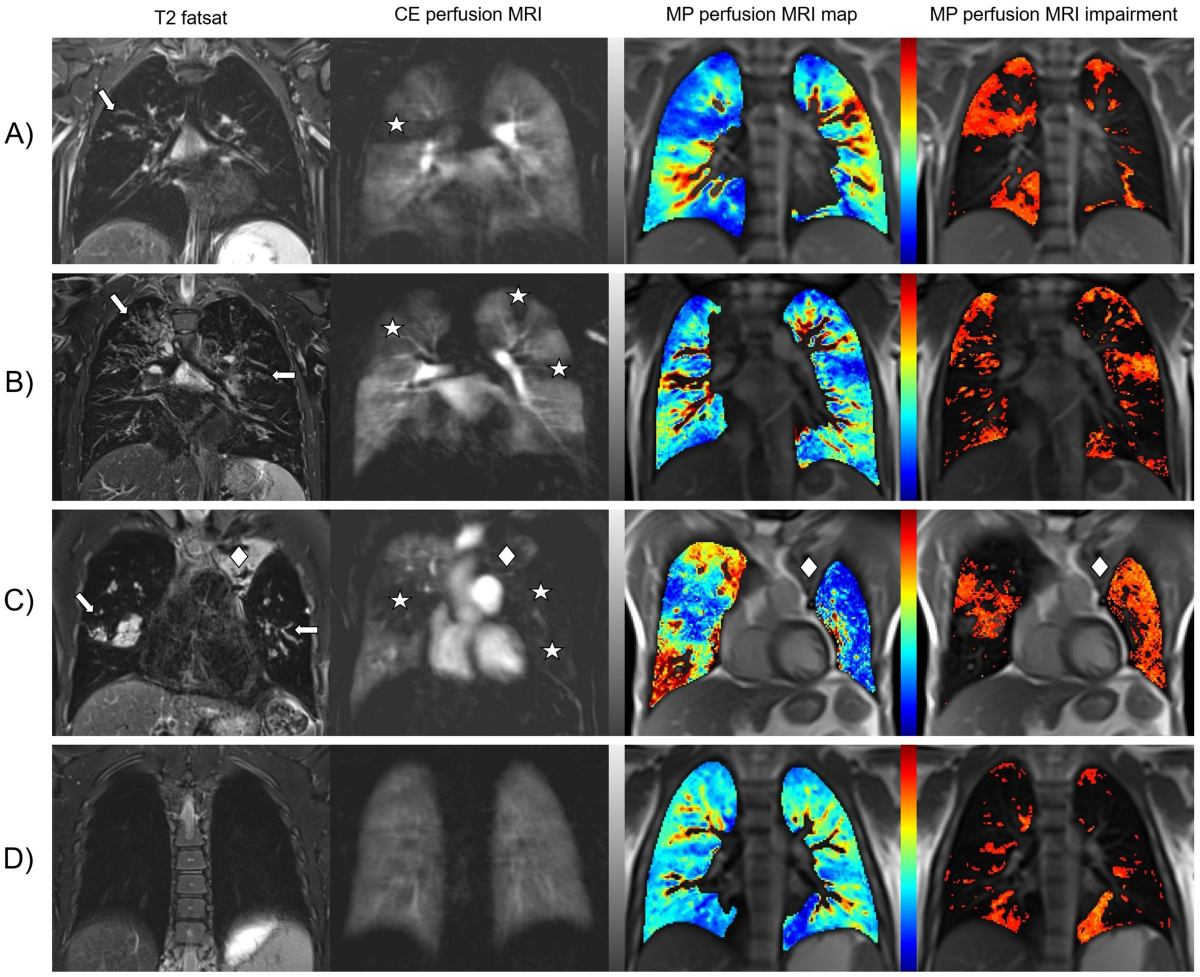
CF-related changes in lung morphology and perfusion detected by morpho-functional MRI and MP-MRI. Representative images of four pwCF with different degrees of lung disease severity. Fat-saturated T2-weighted MRI shows dilated and/or wall-thickened bronchi (arrows). Contrast-enhanced perfusion MRI and color-coded lung maps of MP-MRI show areas of pulmonary hypoperfusion that match with the location of the morphological findings (asterisks). In the gray-scale images of CE perfusion MRI, healthy lung parenchyma with normal Gadolinium uptake appears white to light gray and hypoperfused lung parenchyma is dark gray to black. In the color-coded MP perfusion MRI maps, hypoperfusion is displayed in shades of blue at the lower end of the color scale – the darker the more severe. To simplify visual evaluation, additional impairment maps in shades of red have been calculated to show hypoperfusion only. **(A)** Shows an eight-year-old schoolgirl with intermediate disease severity. For the right upper lobe, all reviewers scored 1 point for bronchiectasis/wall thickening and half of the reviewers scored 1 or 2 points each for CE perfusion and MP perfusion. **(B)** Shows a 20-year-old man with asymmetrically pronounced findings. All reviewers scored 2 points for bronchiectasis/wall thickening, CE perfusion and MP perfusion for the right upper lobe and 1 point each for the left upper lobe. **(C)** Shows a 38-year-old man with advanced CF lung disease. All reviewers scored 1 point for CE perfusion and MP perfusion in the right upper lobe and 2 points each for all three left lobes, as the left lung showed almost complete hypoperfusion. However, this is the only pwCF in the entire study in whom a paramediastinal partial atelectasis of the left upper lobe (rhombus), which was scored as consolidation by all reviewers with 1 point, was not recognized as lung volume by the MP-MRI analysis software “TrueLung.” **(D)** Shows a teenager with only minor lung changes. The most common problem with the MP-MRI visualizations was that all reviewers scored both lower lobes for CE perfusion with no point, while the majority (7/9) scored at least one point for the left lower lobe for MP perfusion. Presumably, heart movements merely simulate these pulmonary hypoperfusions.

**Figure 2:**
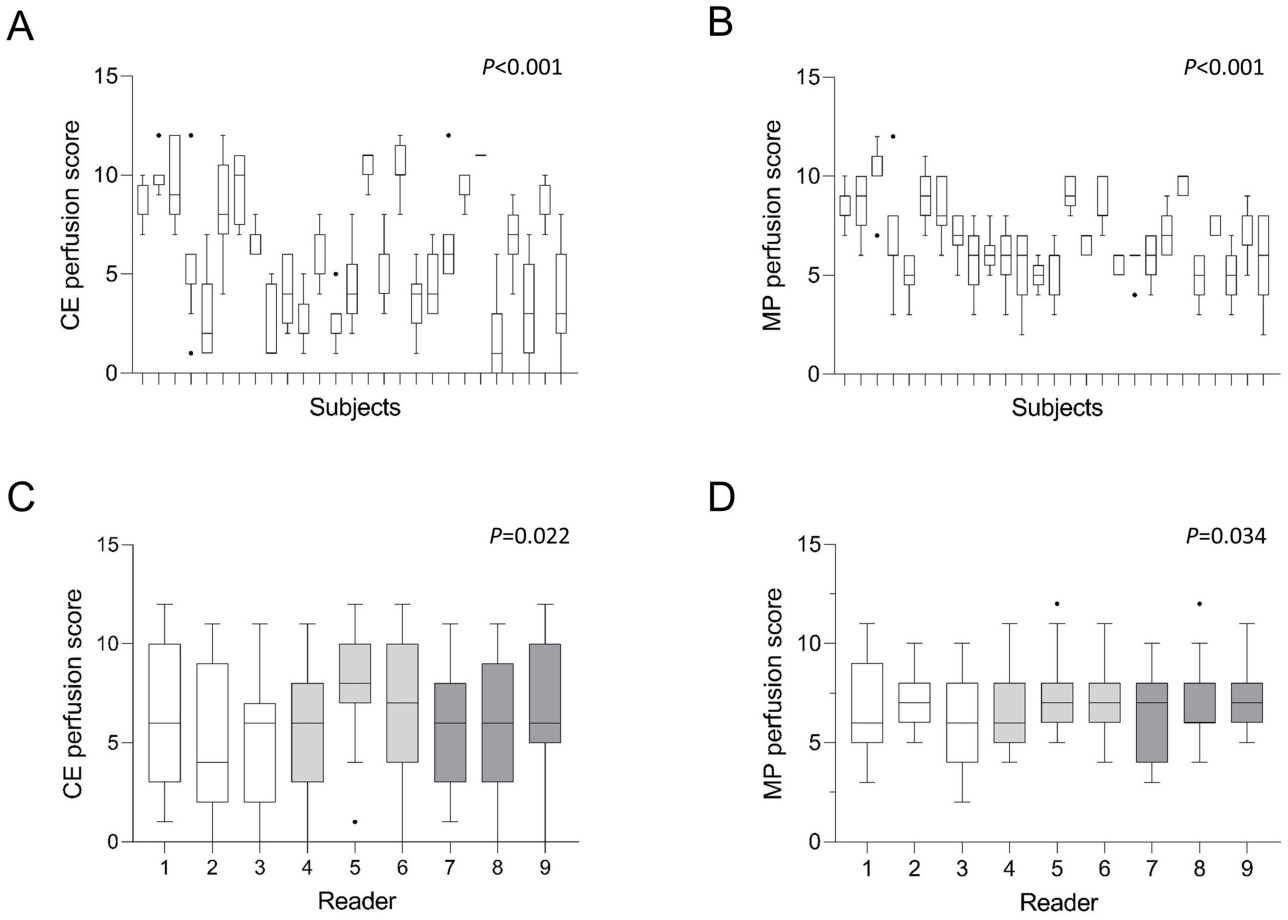
**(A–D)** Mean MRI perfusion scores by subjects and readers. Tukey-Boxplot. The box marks the upper and lower quartiles with the median as the central line. The whiskers represent the maximum and minimum and the dots the outliers. CE = contrast-enhanced, MP = matrix pencil. Kruskal-Wallis test **(A,B)**, Friedman-test **(C,D)**. White bars represent readers with little radiological experience, grey bars readers with intermediate experience and dark grey bars readers with longstanding experience in thoracic radiology. *p* < 0.05 is considered statistically significant.

In the blinded visual imaging analysis, the mean CE perfusion score was 6.2 ± 3.3 and the MP perfusion score was 6.9 ± 2.0 (Table 2; Figure 1). The range of scores varied between a CE perfusion score of 0 and 12 with a median of 6 and a variance of 11.1. The MP perfusion score was rated between 2 and 12 with a median of 7 and a variance of 4.0. The CE perfusion scores were overall lower than the MP perfusion scores (*p* < 0.05). There was a significant difference between CE and MP perfusion scores between the pwCF (both *p* < 0.001) (Figure 2). The respective CE perfusion scores (*p* = 0.022) and MP perfusion scores (*p* = 0.034) differed significantly between the nine readers (Figure 2).

**Table 2:** Mean scores in CE perfusion score, MP perfusion score and morphology score by readers’ experience level.

	Total	Low experience level (*n* = 3)	Intermediate experience level (*n* = 3)	High experience level (*n* = 3)
CE perfusion score	6.22 (3.34)	5.70 (3.41)	6.78 (3.38)	6.19 (3.18)
MP perfusion score	6.89 (1.99)	6.70*** (2.14)	7.17 (1.82)	6.80* (2.00)
Morphology score	15.39 (8.23)	14.32 (7.58)	15.90 (9.13)	15.94 (7.90)

CE, contrast-enhanced-perfusion scores; MP, matrix pencil decomposition. Data are presented as mean ± standard deviation. *P < 0.05, ***P < 0.001 vs. CE perfusion score.

The CE perfusion scores showed strong correlation with the MP perfusion scores (*r* = 0.74, *p* < 0.01). To compare the agreement of scores between the nine readers, the ICC was calculated for each respective score (Table 3). The ICC between the readers showed a good to moderate inter-reader reliability for both MRI perfusion scores (Table 3). It was slightly higher for the CE perfusion score (0.75, 95% confidence interval (CI) 0.63–0.85, *p* < 0.001) than for the MP perfusion score (0.61, CI 0.47–0.75, *p* < 0.001) and significant in both cases.

**Table 3:** Inter-reader agreement (Intraclass correlations (ICC)) of CE perfusion score, MP perfusion score and morphology score in pwCF between readers and by readers’ experience level.

Parameter	All readers		Low experience level	Intermediate experience level	High experience level
	ICC	CI 95%	ICC	ICC	ICC
CE perfusion score	0.75***	0.63–0.85	0.80***	0.59***	0.82***
MP perfusion score	0.61***	0.47–0.75	0.64***	0.57***	0.58***
Morphology score	0.84***	0.76–0.91	0.83***	0.83***	0.86***
Bronchiectasis / wall thickening subscore	0.74***	0.62–0.85	0.66***	0.69***	0.89***
Mucus plugging subscore	0.70***	0.57–0.82	0.68***	0.59***	0.68***
Special findings subscore	0.47***	0.32–0.64	0.29***	0.34***	0.51***
Abscesses / sacculations subscore	0.18***	0.08–0.34	0.11***	0.14***	0.19***
Consolidations subscore	0.79***	0.69–0.88	0.83***	0.77***	0.80***

ICC, intraclass correlation; CI, confidence interval; pwCF, people with cystic fibrosis; CE, contrast-enhanced-perfusion score; MP, matrix pencil decomposition. ***p < 0.001.

For both MRI perfusion scores, the exact agreement, meaning the identical score given as percentage, can be compared. Of the 9 readers rating 27 subjects, 3.7% of the CE perfusion scores were exactly identical. The agreement stayed at 3.7% in case of a tolerance of 1 point and increased to 14.8% with a tolerance of 2 points. Regarding the MP perfusion score, none of the scores was exactly identical. With a tolerance of 1 point, 14.8% of the readers` scores agreed while a 25.9% of the scores overlapped with a tolerance of 2 points. The agreement of perfusion scores between the readers was consequently slightly better in MP-MRI than in CE-MRI.

### Systematic difference between CE and MP perfusion scores

**Figure 3:**
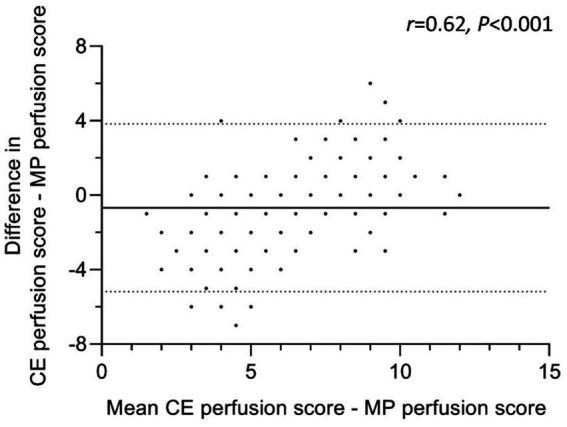
Agreement of CE perfusion score with MP perfusion score. Bland–Altman Plot of 27 people with cystic fibrosis scored by nine readers. Differences between CE perfusion and MP perfusion scores were calculated as absolute differences. The continuous line represents the mean difference and the dashes lines the upper and lower 95% limits of agreement (mean difference ± 1,96*SD). Identical scoring leads to overlapping data points. CE = contrast-enhanced, MP = matrix pencil decomposition. Correlation coefficient *r* describes the relationship between the difference of CE and MP perfusion scores (y-axis) and their mean (x-axis). *p* < 0.05 is considered statistically significant.

To compare the agreement between both perfusion scoring methods, a Bland–Altman analysis was conducted (Figure 3). The absolute difference between CE perfusion scores and MP perfusion scores was correlated with the mean of both perfusion scores. The correlation coefficient *r* was moderate, but significant (*r* = 0.62, *p* < 0.001). Therefore, the lower or the higher the mean perfusion score, the larger the difference between CE and MP perfusion scores (Figure 3). The range of CE perfusion scores assigned by the readers was larger (0–12 points) than the range of MP perfusion scores (2–12 points). With a higher inter-subject-difference in CE perfusion scores, MP perfusion scores were rated higher than the lowest CE perfusion scores and were scored lower than CE perfusion scores at higher mean scores (Figure 3).

### No difference between levels of radiological experience

**Figure 4:**
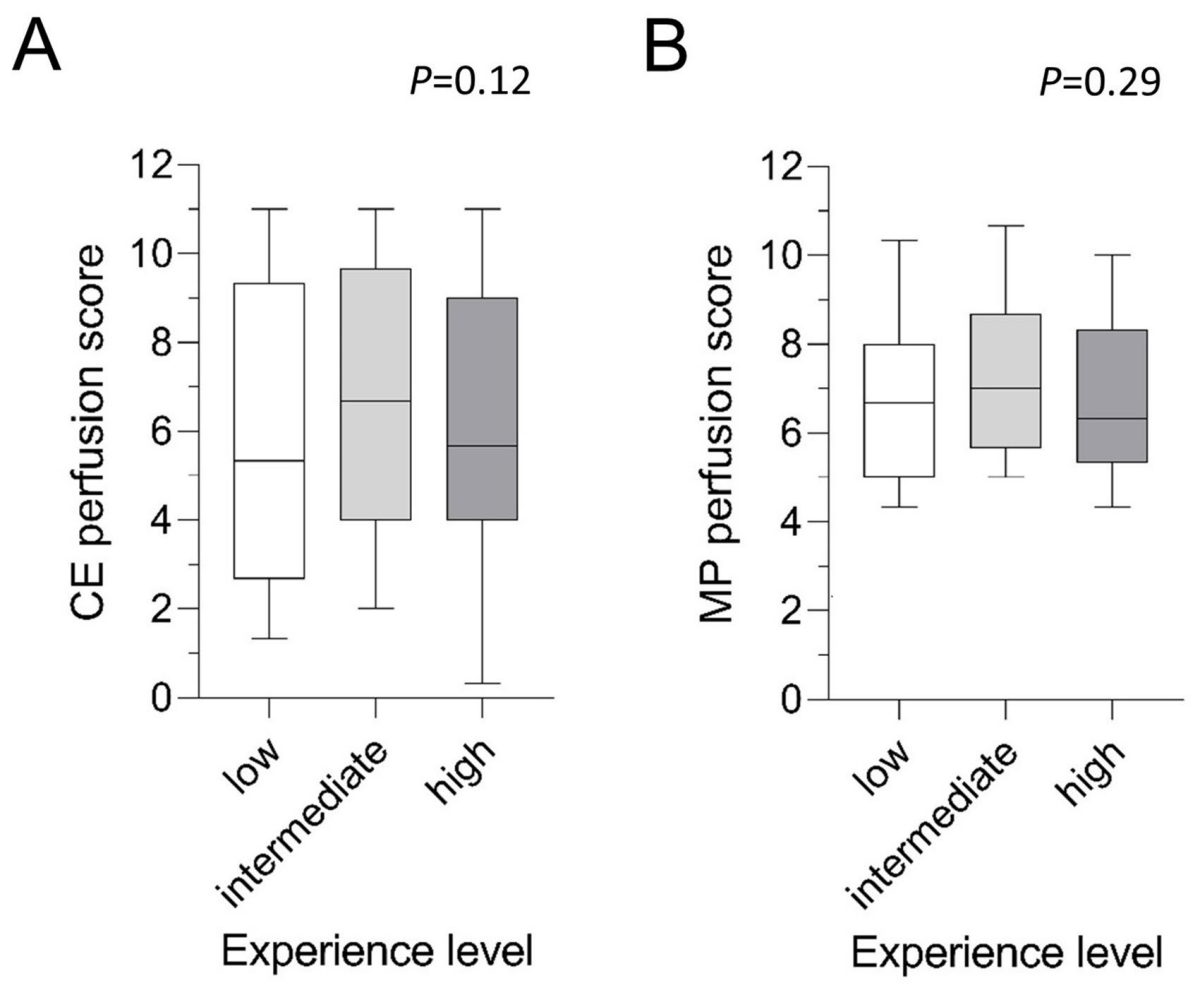
**(A,B)** Mean MRI perfusion scores by experience level. Tukey-Boxplot. The box marks the upper and lower quartiles with the median as the central line. The whiskers represent the maximum and minimum. CE, contrast-enhanced; MP, matrix pencil decomposition. Friedman-test.

The nine readers were summarized into three even-sized groups according to their level of radiological experience. The least experienced readers assigned a mean CE perfusion score of 5.7 ± 3.4 and a mean MP perfusion score of 6.7 ± 2.1 (*p* < 0.001) (Table 2). Intermediate experienced radiologists scored a mean of 6.8 ± 3.4 for CE perfusion and 7.2 ± 1.8 for MP perfusion scores (*p* = 0.12) while chest imaging experts assigned a mean of 6.2 ± 3.2 to CE perfusion and 6.8 ± 2.0 to MP perfusion scores (*p* = 0.018). Comparing the mean scores according to the three experience levels, no significant difference in CE perfusion (*p* = 0.12) and MP perfusion scores (*p* = 0.29) was found (Figure 4). The inter-reader correlation coefficient was significant and moderate to good in all groups and ranged between 0.59–0.82 for CE perfusion scores and 0.57–0.64 for MP perfusion scores (all *p* < 0.001) (Table 3).

### Good agreement of morphological scores between readers and experience levels

**Figure 5:**
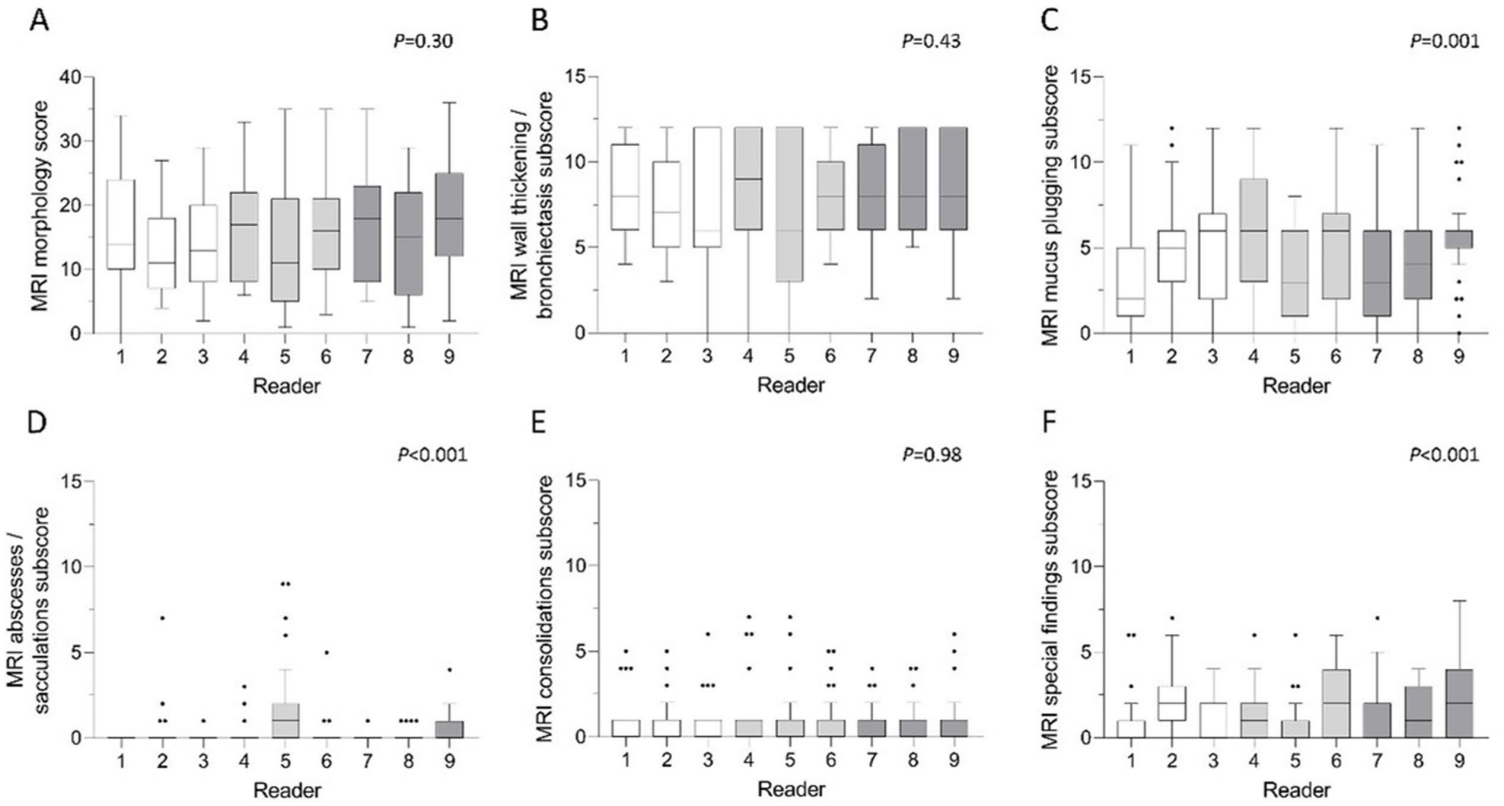
**(A–F)** Mean scores of morphological parameters per reader. **(A)** MRI morphology score, **(B)** bronchiectasis/wall thickening subscore, **(C)** mucus plugging subscore, **(D)** abscesses/sacculations subscore, **(E)** consolidations subscore and **(F)** special findings subscore. White bars represent readers with little radiological experience, grey bars readers with intermediate experience and dark grey bars readers with longstanding experience in thoracic radiology. Friedman-test. *p* < 0.05 is considered statistically significant.

Morphological findings included the following parameters according to the validated chest MRI score: bronchiectasis/wall thickening, mucus plugging, abscesses/sacculations, consolidation and pleural reactions (special findings) (7). The mean MRI morphology score in CE-MRI was 15.4 ± 8.2 in the total cohort (Table 2). The subjects displayed different extents of morphological abnormalities represented by the respective MRI morphology score (*p* < 0.001). However, there was no significant difference between the readers (*p* = 0.30) and the three experience groups (*p* = 0.36) (Figure 5). The ICC showed a strong inter-reader agreement of the morphology score (ICC = 0.84, CI 0.76–0.91, *p* < 0.001). Of the respective subscores, the most frequent pathological finding bronchiectasis/wall thickening displayed a good inter-reader reliability (ICC = 0.74, 0.62–0.85, *p* < 0.001) (Table 3). The inter-reader agreement was similarly good for the mucus plugging subscore (ICC = 0.70, CI 0.57–0.82, *p* < 0.001) as well as the consolidation subscore (ICC = 0.79, CI 0.69–0.88, *p* < 0.001). The special findings subscore (ICC = 0.47, CI 0.32–0.64, *p* < 0.001) and the abscesses/sacculations subscore did not show good agreement between the readers (ICC = 0.18, CI 0.08–0.34, *p* < 0.001) (Table 3). The inter-reader agreement was comparable between the three experience groups and mostly highest in the most experienced group (Table 3).

### Both MRI perfusion scores correlate with the morphological findings showing a stronger correlation in CE perfusion than MP perfusion

**Figure 6:**
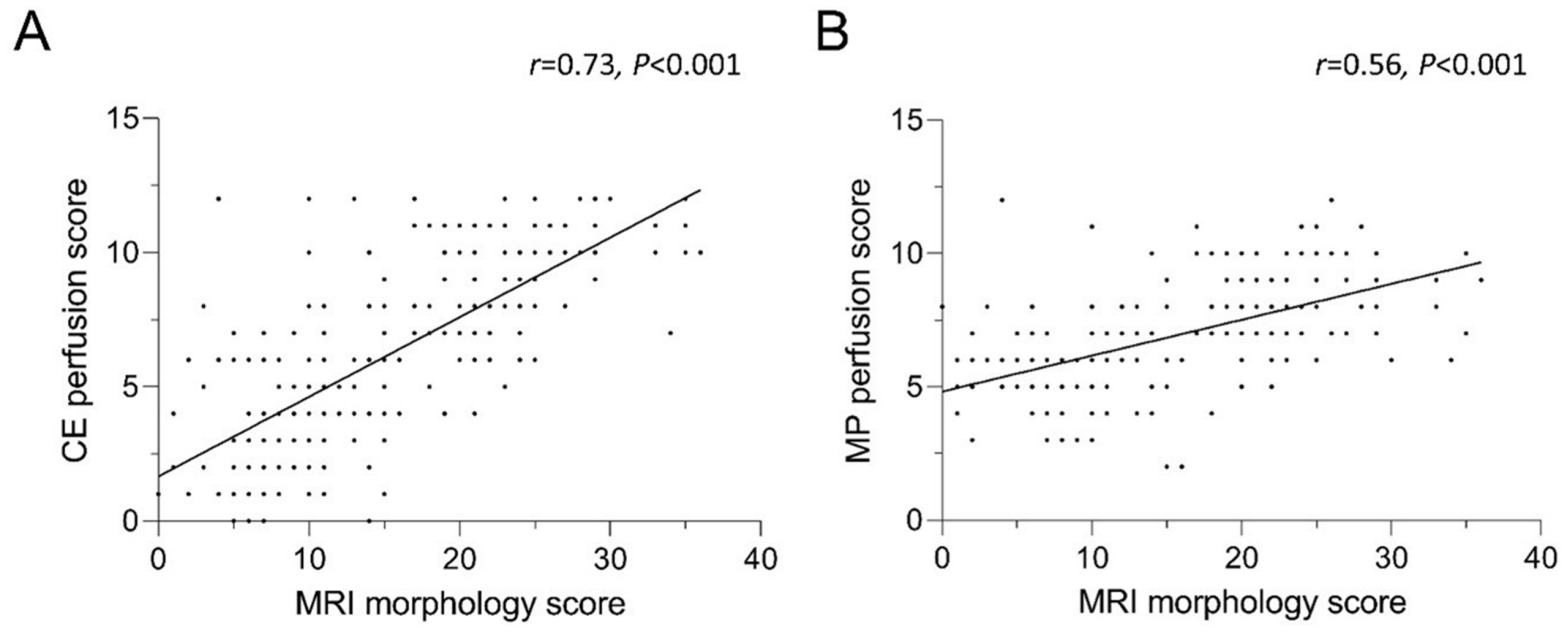
**(A,B)** Correlation between **(A)** CE and **(B)** MP perfusion score and the MRI morphology score. Identical scoring leads to overlapping data points. CE = contrast-enhanced, MP = matrix pencil decomposition. Spearman’s rank correlation coefficient r and *p*-values are provided for each correlation.

To examine the relationship of functional abnormalities and morphological findings, the CE perfusion scores and the MP perfusion scores were correlated with the morphological scores. The morphology score showed a good correlation with the CE perfusion score (*r* = 0.73, *p* < 0.001) (Figure 6). The MP perfusion score significantly correlated with the morphological abnormalities, although with a lower correlation coefficient than the CE perfusion score (*r* = 0.55, *p* < 0.001). The degree of morphological abnormalities consequently increased with higher MRI perfusion scores. This relationship is found for the low experience group (*r* = 0.67, *p* < 0.01; *r* = 0.61, *p* < 0.01), the intermediate experience group (*r* = 0.59, *p* < 0.01; *r* = 0.51, *p* < 0.01) and the high experience group (*r* = 0.74, *p* < 0.01; *r* = 0.57, *p* < 0.01).

## Discussion

This study demonstrates the feasibility of visual perfusion scoring in established contrast-enhanced (CE-) MRI and novel contrast agent-free MP-MRI in pwCF with different degrees of CF lung disease severity. It shows a good inter-reader agreement between the traditional CE-MRI and the alternative contrast agent-free MP-MRI independent of the respective radiologists’ experience level. Scoring of CE-MRI differs from the new MP-MRI method as the CE perfusion scores are generally distributed across a wider range than MP perfusion scores. In both methods, the perfusion scores correlated well with the morphological abnormalities.

First, the absolute CE and MP perfusion scores were compared. This analysis showed slightly higher MP perfusion scores than CE perfusion scores, but a good correlation between both perfusion scores. The inter-reader reliability was good to moderate for both methods but was overall higher for CE perfusion scores. In particular, we observed that the two visual perfusion scores differed in patients with only mild disease severity. In our cohort, several CE perfusion MRIs were scored as 0 points, indicating normal lung perfusion, but all radiologists always scored at least one point in the visual assessment of the MP-MRI lung maps. We explain this by the fact that all radiologists were more used to gray-scaled contrast-enhanced images (CE perfusion) as part of their daily routine in contrast to novel color-coded lung maps (MP perfusion). Physiological small areas of hypoperfusion, e.g., along the pulmonary fissures, were therefore probably more often correctly recognized and less often incorrectly scored as CF-associated. This might explain the slight difference in the intraclass correlation (ICC) between the two methods and therefore the slightly higher ICC in CE perfusion scores. Although all radiologists had undergone initial training, none of the radiologists had any notable routine in image analysis and in scoring of the MP-MRI lung maps. In several patients, there were indications that movement of the beating heart could cause artifacts in the inferior lung segments, especially in the lingula, which could then merely simulate focal hypoperfusion. In panel D of Figure 1, such a case is demonstrated. This is not an unacceptable weakness of the novel method, but the reviewers of MP-MRI lung maps will of course have to gain more experience with these cases. It could furthermore be speculated, that MP-MRI images could potentially be over-simplified and the thicker layers on MP-MRI may have lead to a ‘partial volume effect’. Moreover, the slightly lower spatial resolution of MP-MRI might not represent all perfusion inhomogeneities in detail. As this is the first study to directly compare both approaches, further studies are needed to challenge this hypothesis.

To compare the well-established CE-MRI and the novel MP-MRI method, a Bland–Altman analysis was conducted. The results underline a difference between the two MRI perfusion scoring systems. The data show a wider range of scores given in the CE-MRI compared to MP-MRI. This can be stated for both extremes – intraindividual CE perfusion scores are generally lower or higher than the matching MP perfusion score. From our point of view, the most likely explanation for this result of the study is that the radiological reviewers have significantly more experience with CE perfusion MRI and its visualization in gray-scale images. To simplify the visual analysis of the MP-MRI data, the readers were provided with a “impairment map” in addition to the original MP perfusion MRI map, which was designed to show only the pulmonary hypoperfusion in shades of red (Figure 1). We can only speculate as to whether this graphical representation simplified the visual evaluation to such an extent that our reviewers assessed a smaller range of perfusion scores in MP-MRI. The authors are of the opinion that at this point it cannot even be determined whether this is a strength or weakness of the novel technique. In any case, further studies are needed to confirm this observation and to investigate its cause. Therefore, a more extensive routine in the interpretation of these novel images and a cross-validation with contrast-enhanced chest MRI should be established. The difference between the functional methods could potentially be overcome by an automated analysis which standardizes the assessment and can be implemented reader-independently in different centers and therefore enables international harmonization and reproducibility. To investigate parts of this approach, our research group was recently able to demonstrate a strong correlation between CE-MRI and automatically processed MP-MRI in another, independent study (36). First studies have furthermore included semi-automated analysis and support by machine learning approaches (20, 32, 36, 40). These developments are promising to propel international standardization, but are not yet fully developed for all radiological features.

We were furthermore interested in the impact of the readers’ radiological experience on the assigned MRI scores. The comparison of the nine readers with different degrees of experience demonstrated a significant difference between the readers’ scoring but no difference that could be attributed to the radiological experience. So far, very few studies have compared the impact of different radiological experience levels on the evaluation of chest MRI. Most existing studies have compared readers of similar experience or validated different radiological methods by multireader approaches. Nauck et al. (33) analyzed same-day MRI and CT in patients with COPD and three readers with a good concordance between the methods and lower inter-reader agreement on MRI than on CT. Similarly, another study group reported a fair to moderate inter-reader concordance to detect pulmonary ground glass opacities and fibrosis-like changes on MRI and CT with a higher inter-reader agreement on CT than on MRI (27). As mentioned previously, this may be well understood by the decreased spatial resolution of MRI. Willers et al. (32) studied intra-reader repeatability and inter-observer reproducibility as well as the impact of a trained neural network in MP-MRI in ten healthy and 25 children with CF. The reported intra-reader repeatability was very good with still a strong, but not perfect inter-observer reproducibility. A more heterogeneous study group and more diverging experience levels of the readers in our study might explain the higher inter-reader agreement by Willers et al. (32). However, our conclusions are consistent with their findings reporting systematic differences between readers and a potential benefit through automation procedures. To compare ratings of ventilation impairment and ventilation defect scores on ^129^Xe-MRI, Ebner et al. (28) analyzed results by five blinded readers with a comparable degree of experience. Overall inter-reader agreement was substantial with kappa coefficient of 0.7. Depending on the lung region, some ratings varied markedly, leading to a kappa coefficient between 0.4 and 0.7. The described inter-reader agreement was comparable to our findings and might similarly be influenced by a heterogeneous study cohort and a decreased spatial resolution (32). Most studies underline the positive impact of high radiological experience on the outcome parameter and a higher sensitivity to detect abnormalities (29, 41). The impact of the radiologists’ experience may however differ depending on the outcome parameter and the applied radiological method (30, 31, 42).

This study reports a significant correlation between morphological findings summarized in the morphology score and the CE- and MP perfusion scores, whereas the correlation with CE perfusion was higher than for MP perfusion scores. The correlation between structural and functional abnormalities in the CF lung is comprehensible as worsening perfusion impairment is usually related to the progression of structural pathologies such as mucus plugging, consolidations or sacculations (17). As the present study cohort shows rather heterogeneous lung disease severity, the association between morphological and functional abnormalities does not seem to be restricted to a certain level of disease. These results are in line with findings by Doellinger et al. and other study groups (20, 36, 40).

The objective of our study was to compare the established CE-MRI with novel MP-MRI techniques to estimate potential comparability and use of MP-MRI as contrast agent-free method in future. In this context, these two MRI-based approaches were examined without the comparison with other clinical measures. However, a preceding study from our group has shown good correlation of both approaches with spirometry and therefore the ability of MP-MRI to identify disease severity (36). Other research groups have evaluated the reproducibility of MP-MRI in healthy children and individuals with CF as important prerequisite for the successful clinical application (43). A swiss study group has shown the correlation between CE- and MP-MRI with other lung function methods such as multiple-breath washout (MBW) (20, 40). In young children or generally milder CF lung disease, MBW is more sensitive to detect ventilation impairments than spirometry (40). Based on results showing MRI sensitivity to detect pulmonary disease and its progression, first studies have additionally demonstrated capability of MP-MRI to capture response to treatment (20, 44). Therefore, these findings support the use of MRI for chest examination in multicenter studies and the clinical routine care in pwCF of all ages. Importantly, the sensitivity of MP-MRI to detect perfusion inhomogeneities allows contrast agent-free examinations in future and might render intravenous contrast application obsolete. A non-invasive MRI exam not only simplifies the procedure for the medical facility, it also increases patient comfort as well as patient safety, since possible undesirable effects of gadolinium-based contrast agents are avoided. In our center, the acquisition of MP-MRI has been part of the standard MRI protocol for pwCF since 2020. Our patients and their parents constantly assure us that they are happy to accept the significantly longer examination time (MP-MRI 6–8 min vs. CE-MRI <1 min) if this prevents from venipuncture and the long breath hold of 45 s, which can be very long for pwCF. The avoidance of i.v. contrast application is especially important for patients allergic to contrast agents, those with renal failure or other contraindications and patient groups for which specific contrast agents are not approved. Considering potential permanent intracorporal deposits, especially with the use of gadolinium-based contrast media, the contrast-agent free functional imaging by MP-MRI might be a promising alternative in future.

One limitation of this study is the rather small sample size of 27 pwCF. Lastly, despite defined criteria, the scoring of images is still a subjective task. Therefore, the scores differ between the readers and an objective criterion to verify the correct identification of pathologies or a different examination method to compare with has not been used in this study. Therefore, the readers’ ratings can only be compared with each other but no other reference measure to confirm findings.

In summary, our study in 27 pwCF shows slightly higher MP perfusion scores compared with CE perfusion scores, but a good inter-reader reliability for both perfusion scores. The CE perfusion scores showed a larger variability and a systematic difference compared to MP perfusion scores especially in very low and high scores which may be due to the readers’ routine with gray-scale CE perfusion images rather than novel color-coded lung maps of MP-MRI. The degree of radiological experience did not impact the results, but the investigated differences between human readers could be reduced by an automated, reader-independent analysis. Both MRI perfusion methods correlated well with morphological abnormalities in this heterogenous CF group with different CF lung disease severity levels. The novel MP-MRI holds promise to facilitate contrast agent-free and therefore non-invasive quantitative studies of abnormalities in lung perfusion in pwCF. It will hopefully advance the development of novel, sensitive outcome measures in lung disease research, multicenter studies and the clinical routine care.

## Data Availability

The raw data supporting the conclusions of this article will be made available by the authors, without undue reservation.

## References

[1] Shteinberg M Haq IJ Polineni D Davies JC. Cystic fibrosis. Lancet. (2021) 397:2195–211. doi: 10.1016/S0140-6736(20)32542-3, PMID: 34090606

[2] Mall MA Burgel PR Castellani C Davies JC Salathe M Taylor-Cousar JL. Cystic fibrosis. Nat Rev Dis Primers. (2024) 10:53. doi: 10.1038/s41572-024-00538-6, PMID: 39117676

[3] Grasemann H Ratjen F. Cystic fibrosis. N Engl J Med. (2023) 389:1693–707. doi: 10.1056/NEJMra2216474, PMID: 37913507

[4] Elborn JS. Cystic fibrosis. Lancet. (2016) 388:2519–31. doi: 10.1016/S0140-6736(16)00576-6 27140670

[5] Montgomery ST Mall MA Kicic A Stick SM. Hypoxia and sterile inflammation in cystic fibrosis airways: mechanisms and potential therapies. Eur Respir J. (2017) 49:1600903. doi: 10.1183/13993003.00903-2016, PMID: 28052955

[6] Goralski JL Stewart NJ Woods JC. Novel imaging techniques for cystic fibrosis lung disease. Pediatr Pulmonol. (2021) 56:S40–s54. doi: 10.1002/ppul.24931, PMID: 32592531 PMC7808406

[7] Eichinger M Optazaite DE Kopp-Schneider A Hintze C Biederer J Niemann A. Morphologic and functional scoring of cystic fibrosis lung disease using MRI. Eur J Radiol. (2012) 81:1321–9. doi: 10.1016/j.ejrad.2011.02.045, PMID: 21429685

[8] Eichinger M Heussel CP Kauczor HU Tiddens H Puderbach M. Computed tomography and magnetic resonance imaging in cystic fibrosis lung disease. JMRI. (2010) 32:1370–8. doi: 10.1002/jmri.22374 21105141

[9] Puderbach M Eichinger M Haeselbarth J Ley S Kopp-Schneider A Tuengerthal S. Assessment of morphological MRI for pulmonary changes in cystic fibrosis (CF) patients: comparison to thin-section CT and chest x-ray. Investig Radiol. (2007) 42:715–24. doi: 10.1097/RLI.0b013e318074fd81, PMID: 17984769

[10] Wielputz MO von Stackelberg O Stahl M Jobst BJ Eichinger M Puderbach MU. Multicentre standardisation of chest MRI as radiation-free outcome measure of lung disease in young children with cystic fibrosis. J Cyst Fibros. (2018) 17:518–27. doi: 10.1016/j.jcf.2018.05.003, PMID: 29805050

[11] Stahl M Steinke E Graeber SY Joachim C Seitz C Kauczor HU. Magnetic resonance imaging detects progression of lung disease and impact of newborn screening in preschool children with cystic fibrosis. Am J Respir Crit Care Med. (2021) 204:943–53. doi: 10.1164/rccm.202102-0278OC, PMID: 34283704

[12] Stahl M Roehmel J Eichinger M Doellinger F Naehrlich L Kopp MV. Effects of Lumacaftor/Ivacaftor on cystic fibrosis disease progression in children 2 through 5 years of age homozygous for F508del-CFTR: a phase 2 placebo-controlled clinical trial. Ann Am Thorac Soc. (2023) 20:1144–55. doi: 10.1513/AnnalsATS.202208-684OC, PMID: 36943405 PMC10405608

[13] Stahl M Wielpütz MO Ricklefs I Dopfer C Barth S Schlegtendal A. Preventive inhalation of hypertonic saline in infants with cystic fibrosis (PRESIS). A randomized, double-blind, controlled study. Am J Respir Crit Care Med. (2019) 199:1238–48. doi: 10.1164/rccm.201807-1203OC 30409023

[14] Stahl M Dohna M Graeber SY Sommerburg O Renz DM Pallenberg ST. Impact of Elexacaftor/Tezacaftor/Ivacaftor therapy on lung clearance index and magnetic resonance imaging in children with cystic fibrosis and one or two F508del alleles. Eur Respir J. (2024) 64:2400004. doi: 10.1183/13993003.00004-2024, PMID: 38901883 PMC11375515

[15] Heidenreich JF Kuhl PJ Grunz JP Hendel R Metz C Weng AM. Lung function in patients with cystic fibrosis before and during CFTR-modulator therapy using 3D ultrashort Echo time MRI. Radiology. (2023) 308:e230084. doi: 10.1148/radiol.230084, PMID: 37404154

[16] Graeber SY Renz DM Stahl M Pallenberg ST Sommerburg O Naehrlich L. Effects of Elexacaftor/Tezacaftor/Ivacaftor therapy on lung clearance index and magnetic resonance imaging in patients with cystic fibrosis and one or two F508del alleles. Am J Respir Crit Care Med. (2022) 206:311–20. doi: 10.1164/rccm.202201-0219OC, PMID: 35536314

[17] Wielputz MO Puderbach M Kopp-Schneider A Stahl M Fritzsching E Sommerburg O. Magnetic resonance imaging detects changes in structure and perfusion, and response to therapy in early cystic fibrosis lung disease. Am J Respir Crit Care Med. (2014) 189:956–65. doi: 10.1164/rccm.201309-1659OC, PMID: 24564281

[18] Klimeš F Voskrebenzev A Gutberlet M Obert AJ Pöhler GH Grimm R. Repeatability of dynamic 3D phase-resolved functional lung (PREFUL) ventilation MR imaging in patients with chronic obstructive pulmonary disease and healthy volunteers. JMRI. (2021) 54:618–29. doi: 10.1002/jmri.27543, PMID: 33565215

[19] Voskrebenzev A Gutberlet M Klimeš F Kaireit TF Schönfeld C Rotärmel A. Feasibility of quantitative regional ventilation and perfusion mapping with phase-resolved functional lung (PREFUL) MRI in healthy volunteers and COPD, CTEPH, and CF patients. Magn Reson Med. (2018) 79:2306–14. doi: 10.1002/mrm.26893, PMID: 28856715

[20] Streibel C Willers CC Pusterla O Bauman G Stranzinger E Brabandt B. Effects of elexacaftor/tezacaftor/ivacaftor therapy in children with cystic fibrosis - a comprehensive assessment using lung clearance index, spirometry, and functional and structural lung MRI. J Cyst Fibros. (2023) 22:615–22. doi: 10.1016/j.jcf.2022.12.012, PMID: 36635199

[21] Bauman G Bieri O. Matrix pencil decomposition of time-resolved proton MRI for robust and improved assessment of pulmonary ventilation and perfusion. Magn Reson Med. (2017) 77:336–42. doi: 10.1002/mrm.26096, PMID: 26757102

[22] Stahl M Wielputz MO Graeber SY Joachim C Sommerburg O Kauczor HU. Comparison of lung clearance index and magnetic resonance imaging for assessment of lung disease in children with cystic fibrosis. Am J Respir Crit Care Med. (2017) 195:349–59. doi: 10.1164/rccm.201604-0893OC, PMID: 27575911

[23] Bauman G Puderbach M Heimann T Kopp-Schneider A Fritzsching E Mall MA. Validation of Fourier decomposition MRI with dynamic contrast-enhanced MRI using visual and automated scoring of pulmonary perfusion in young cystic fibrosis patients. Eur J Radiol. (2013) 82:2371–7. doi: 10.1016/j.ejrad.2013.08.018, PMID: 24016829

[24] Dohna M Voskrebenzev A Klimeš F Kaireit TF Glandorf J Pallenberg ST. PREFUL MRI for monitoring perfusion and ventilation changes after Elexacaftor-Tezacaftor-Ivacaftor therapy for cystic fibrosis: a feasibility study. Radiol Cardiothorac Imaging. (2024) 6:e230104. doi: 10.1148/ryct.230104, PMID: 38573129 PMC11056757

[25] Veldhoen S Weng AM Knapp J Kunz AS Stäb D Wirth C. Self-gated non-contrast-enhanced functional lung MR imaging for quantitative ventilation assessment in patients with cystic fibrosis. Radiology. (2017) 283:242–51. doi: 10.1148/radiol.2016160355 27715657

[26] Hellgren R Tolocka E Saracco A Wilczek B Sundbom A Hall P. Comparing the diagnostic accuracy, reading time, and inter-rater agreement of breast MRI abbreviated and full protocols: a multi-reader study. Acta Radiol. (2023) 65:195–201. doi: 10.1177/02841851231216552, PMID: 38115682 PMC10903132

[27] Azour L Condos R Keerthivasan MB Bruno M Pandit Sood T Landini N. Low-field 0.55 T MRI for assessment of pulmonary groundglass and fibrosis-like opacities: inter-reader and inter-modality concordance. Eur J Radiol. (2022) 156:110515. doi: 10.1016/j.ejrad.2022.110515, PMID: 36099832 PMC10347896

[28] Ebner L Virgincar RS He M Choudhury KR Robertson SH Christe A. Multireader determination of clinically significant obstruction using hyperpolarized (129)Xe-ventilation MRI. AJR Am J Roentgenol. (2019) 212:758–65. doi: 10.2214/AJR.18.20036, PMID: 30779661 PMC7079551

[29] Pesapane F Nicosia L Tantrige P Schiaffino S Liguori A Montesano M. Inter-reader agreement of breast magnetic resonance imaging and contrast-enhanced mammography in breast cancer diagnosis: a multi-reader retrospective study. Breast Cancer Res Treat. (2023) 202:451–9. doi: 10.1007/s10549-023-07093-w, PMID: 37747580

[30] Min JH Lee MW Park HS Lee DH Park HJ Lim S. Interobserver variability and diagnostic performance of Gadoxetic acid-enhanced MRI for predicting microvascular invasion in hepatocellular carcinoma. Radiology. (2020) 297:573–81. doi: 10.1148/radiol.2020201940, PMID: 32990512

[31] Pricolo P Ancona E Summers P Abreu-Gomez J Alessi S Jereczek-Fossa BA. Whole-body magnetic resonance imaging (WB-MRI) reporting with the METastasis reporting and data system for prostate Cancer (MET-RADS-P): inter-observer agreement between readers of different expertise levels. Cancer Imaging. (2020) 20:77. doi: 10.1186/s40644-020-00350-x, PMID: 33109268 PMC7590732

[32] Willers C Bauman G Andermatt S Santini F Sandkühler R Ramsey KA. The impact of segmentation on whole-lung functional MRI quantification: repeatability and reproducibility from multiple human observers and an artificial neural network. Magn Reson Med. (2021) 85:1079–92. doi: 10.1002/mrm.28476, PMID: 32892445

[33] Nauck S Pohl M Jobst BJ Melzig C Meredig H Weinheimer O. Phenotyping of COPD with MRI in comparison to same-day CT in a multi-Centre trial. Eur Radiol. (2024) 34:5597–609. doi: 10.1007/s00330-024-10610-0, PMID: 38345607 PMC11364611

[34] Bauman G Puderbach M Deimling M Jellus V Chefd’hotel C Dinkel J. Non-contrast-enhanced perfusion and ventilation assessment of the human lung by means of fourier decomposition in proton MRI. Magn Reson Med. (2009) 62:656–64. doi: 10.1002/mrm.22031, PMID: 19585597

[35] Bauman G Pusterla O Bieri O. Ultra-fast steady-state free precession pulse sequence for Fourier decomposition pulmonary MRI. Magn Reson Med. (2016) 75:1647–53. doi: 10.1002/mrm.25697, PMID: 25965158

[36] Doellinger F Bauman G Roehmel J Stahl M Posch H Steffen IG. Contrast agent-free functional magnetic resonance imaging with matrix pencil decomposition to quantify abnormalities in lung perfusion and ventilation in patients with cystic fibrosis. Front Med. (2024) 11:1349466. doi: 10.3389/fmed.2024.1349466, PMID: 38903825 PMC11188455

[37] Pusterla O Willers C Sandkühler R Andermatt S Nyilas S Cattin PC. An automated pipeline for computation and analysis of functional ventilation and perfusion lung MRI with matrix pencil decomposition: TrueLung. Z Med Phys. (2024). [online ahead of print]. doi: 10.1016/j.zemedi.2024.08.001, PMID: 39304382 PMC12766494

[38] Karlik SJ. Exploring and summarizing radiologic data. AJR Am J Roentgenol. (2003) 180:47–54. doi: 10.2214/ajr.180.1.1800047, PMID: 12490475

[39] Liljequist D Elfving B Skavberg RK. Intraclass correlation - a discussion and demonstration of basic features. PLoS One. (2019) 14:e0219854. doi: 10.1371/journal.pone.0219854, PMID: 31329615 PMC6645485

[40] Frauchiger BS Willers C Cotting J Kieninger E Korten I Casaulta C. Lung structural and functional impairments in young children with cystic fibrosis diagnosed following newborn screening - a nationwide observational study. J Cyst Fibros. (2024) 23:910–7. doi: 10.1016/j.jcf.2024.05.010 38926017

[41] Di Franco F Souchon R Crouzet S Colombel M Ruffion A Klich A. Characterization of high-grade prostate cancer at multiparametric MRI: assessment of PI-RADS version 2.1 and version 2 descriptors across 21 readers with varying experience (MULTI study). Insights Imaging. (2023) 14:391. doi: 10.1186/s13244-023-01391-z PMC10027981 36939970

[42] van Grinsven S Hagenmaier F van Loon CJ van Gorp MJ van Kints MJ van Kampen A. Does the experience level of the radiologist, assessment in consensus, or the addition of the abduction and external rotation view improve the diagnostic reproducibility and accuracy of MRA of the shoulder? Clin Radiol. (2014) 69:1157–64. doi: 10.1016/j.crad.2014.07.009, PMID: 25218253

[43] Nyilas S Bauman G Pusterla O Ramsey K Singer F Stranzinger E. Ventilation and perfusion assessed by functional MRI in children with CF: reproducibility in comparison to lung function. J Cyst Fibros. (2019) 18:543–50. doi: 10.1016/j.jcf.2018.10.003, PMID: 30348613

[44] Kieninger E Willers C Röthlisberger K Yammine S Pusterla O Bauman G. Effect of salbutamol on lung ventilation in children with cystic fibrosis: comprehensive assessment using spirometry, multiple-breath washout, and functional lung magnetic resonance imaging. Respiration. (2022) 101:281–90. doi: 10.1159/000519751, PMID: 34808631

